# Large Right Atrial Thrombus Associated with Central Venous Catheter Requiring Open Heart Surgery

**DOI:** 10.1155/2012/501303

**Published:** 2012-11-06

**Authors:** Nasir Hussain, Paul Eric Shattuck, Mourad Hussein Senussi, Erwin Velasquez Kho, Mubeenkhan Mohammedabdul, Devang K. Sanghavi, Usman Mustafa, Arvind Balavenkataraman, Dragic M. Obradovic

**Affiliations:** ^1^Department of Internal Medicine, Saint Joseph Hospital, Resurrection Health Care, 2900 North Lake Shore Drive, Chicago, IL 60657, USA; ^2^Division of Cardiology, Saint Joseph Hospital, Resurrection Health Care, Chicago, IL 60657, USA

## Abstract

Central venous catheters (CVC) are used commonly in clinical practice. Incidences of CVC-related right atrial thrombosis (CRAT) are variable, but, when right atrial thrombus is present, it carries a mortality risk of 18% in hemodialysis patients and greater than 40% risk in nonhemodialysis patients. Different pathogenic mechanisms have been postulated for the development of CRAT, which includes mechanical irritation of the myocardial wall, propagation of intraluminal clot, hypercoagulability, and hemodynamics of right atria. Presentation of CRAT may be asymptomatic or may be associated with one of the complications of CRAT like pulmonary embolism, systemic embolism, infected thrombi, or hemodynamic compromise. There are no established treatment guidelines for CRAT. We describe an interesting case of a 59-year-old asymptomatic male successfully treated with open heart surgery after failure of medical treatment for a large CRAT discovered during a preoperative evaluation for a kidney transplant. Our case underscores that early detection of CRAT may carry a favorable prognosis as opposed to waiting until catastrophic complications arise. It also underscores the importance of transesophageal echocardiography in the detection of thrombus and perhaps guides clinicians on which treatment modality to be used according to the size of the thrombus.

## 1. Introduction

Catheter-related right atrial thrombosis (CRAT) is a rare as well as underreported but potentially life threatening complication of central venous catheters (CVC). There are no established medical or surgical treatment guidelines for CRAT, further there are no guidelines regarding choice of treatment setting, whether outpatient or inpatient, in particular if the patient is asymptomatic. In this paper we have reviewed the recently published literature and discussed some of the key factors involved in management of CRAT, like early recognition of CRAT with a possible survival benefit, monitoring in a hospitalized setting, and influence of size of thrombus on the treatment modality.

## 2. Case Presentation

**Figure 1:**
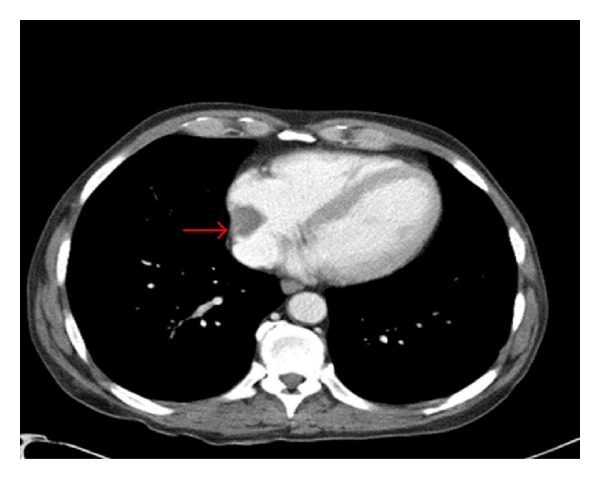
Showing CRAT 2 weeks prior to presentation.

**Figure 2:**
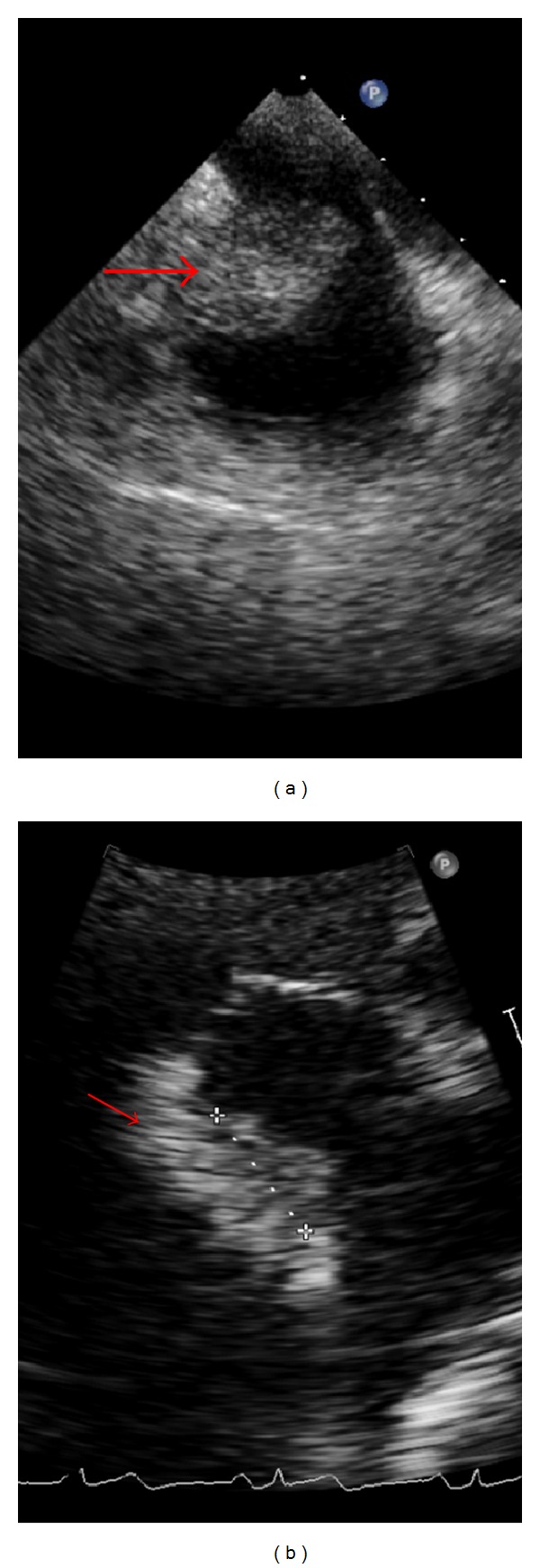
(a) Shows CRAT at presentation (b) shows CRAT after one week of anticoagulation.

A 59-year-old asymptomatic male with end stage renal disease (ESRD) was found to have an ovoid mass in the right atrium on CT scan of abdomen (Figure 1) done as an outpatient preoperative evaluation for kidney transplantation. Considering that the mass may be a clot, the patient had underwent outpatient transthoracic (TTE) followed by transesophageal Echocardiogram (TEE) in our facility, which showed a new 4 cm large right atrial thrombus (RAT) (Figure 2(a)) compared to the TTE done 3 months ago. The thrombus was adherent to the catheter tip as well as the right atrial wall obstructing at least one-third of the right atrial cavity. The patient was subsequently admitted to the intensive care unit for further care. 

The patient had a right kidney transplant for ESRD (10 years prior to presentation) and had a subsequent ESRD due to recently diagnosed (3 months Prior to presentation) Wegener's granulomatosis. The patient had a tunneled double lumen hemodialysis (HD) catheter (15 Fr, silicone rubber) (Interventional Radiology guided Quinton PERM-A-CATH) placed in the right internal jugular vein (central venous catheter, CVC) 3 months prior to presentation wherein the catheter tip had extended all the way to the right atrial wall. 

HD center staff maintained CVC care and reported usage of heparin lock for prevention of catheter thrombosis. There was no history of any recent catheter infection. The patient did not have any history of thrombosis, graft failure in past, and there was no documented evidence of thrombophilia. HD staff had noted increased resistance in CVC flow during the patient's last two hemodialysis (HD) sessions.

At the time of the initial encounter, vitals were stable; physical exam was normal and was negative for any suggestive signs of CRAT. Admission labs including CBC, CMP, PT/INR, and APTT were normal except for evidence of anemia (Hb-11.6), high BUN (54), and serum creatinine (7.69), and platelet count was normal (197). Admission EKG showed some early repolarization changes. 

After consultation with interventional radiology as well as cardiothoracic surgery and in light of the available literature for this type of problem it was decided to treat the patient conservatively with heparin and subsequent oral warfarin unless or until there is a propagation of the mass, failure of the clot to resolve, development of complication, or if the patient becomes symptomatic. A new femoral Quinton catheter was placed for HD and complete avoidance of use of the right CVC. Catheter removal as well as use of thrombolytic therapy was deemed too risky at the time as either may result in pulmonary embolism and add unnecessary morbidity and a risk for mortality. The patient was treated conservatively with anticoagulation and was followed up with serial TTEs. An interval decrease in the size of the thrombus to approximately 3.2 cm (Figure 2(b)) after one week of treatment was noted but was still insufficient in terms of allowing for removal of CVC. 

**Figure 3:**
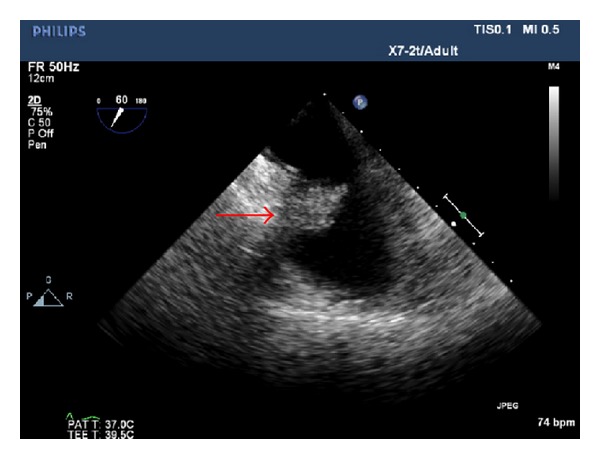
It shows organized thrombus.

Despite of continued anticoagulation for a period of 12 days, the clot became more organized (Figure 3) without any further decrease in the size. After a lengthy discussion, the patient opted for open heart surgery. During surgery, the patient was placed on extracorporeal circulation, right atriotomy was performed, and the right atrial clot along with a portion of the CVC was removed and sent for pathology exam. The patient tolerated surgery well without any complications. The next day the patient underwent interventional radiology guided removal of the remaining portion of CVC. The pathology result confirmed that right atrial mass was a blood clot measuring 3∗2∗0.6 cm. Subsequently, left forearm arteriovenous graft (AVG) for HD was placed; patient was started on Coumadin due to persistent postoperative atrial fibrillation and was discharged to follow up as an outpatient. 

## 3. Discussion

CVC are frequently used in ESRD patients for HD while awaiting AVG placement and maturation. The reported incidence for CRAT is variable 2–29% [1, 2]. Potential mortality associated with RAT has been reported to be as high as 18% in HD patients [3] and greater than 40% in nonhemodialysis patients [3]. There are two types of RAT, type A and B. Type A is mobile, is thromboembolic in nature and has a higher incidence of PE as well as a higher mortality as compared to type B [3]. Type B thrombus forms around foreign bodies in the right atria [3], such as in our case paper. Pathogenesis for type B RAT includes mechanical irritation of the right atrial wall [4], intraluminal clot elongation [5], hypercoagulability in HD patients [6], and fluid dynamics of the right atria [7]. RAT type B may be asymptomatic [3] in presentation as in our case; other associated symptoms may arise from complications of RAT which include pulmonary embolism, systemic embolism, infected thrombi, and hemodynamic compromise [3]. Aforementioned complications may result in a higher mortality in type B RAT [3]. 

CRAT may be found incidentally upon imaging for some other reasons or may be suspected if there are associated symptoms or signs such as resistance in blood flow in CVC noted during HD. Resistance in blood flow in CVC may represent an early sign of RAT, as in our patient. 

CRAT is underreported phenomena because of two folds; first, some patients are entirely asymptomatic at presentation of CRAT [7]; second, diagnostic accuracy of TEE may be limited if catheter tip is proximal in superior vena cava [7]. 

In suspected cases, TEE can be done and TEE has a better sensitivity and specificity compared to TTE [7]. Cardiac MRI with gadolinium contrast can be a useful tool for diagnosis and for tissue characterization but was avoided in our case because of risk of nephrogenic systemic fibrosis [8]. 

Oral/systemic anticoagulation, surgical thrombectomy (open heart, percutaneous), and thrombolysis are reported treatment options for CRAT [3]; all modalities should be combined with removal of CVC after an initial period of anticoagulation [3]. There is no reported survival benefit whether patient is treated medically or surgically [3]. In our case, we proceeded with anticoagulation until there was no further decrease in clot that size was noted at which point the patient underwent surgical thrombectomy. Although the literature shows that some asymptomatic CRAT may be treated successfully with systemic/oral anticoagulation for a period of six months, we postulate that the bigger the size of clot, the greater the chance that medical treatment will fail. Our case supports findings of lower mortality in surgery groups by Negulescu et al. [9] and that thrombi larger than 2 cm should undergo surgical thrombectomy in absence of any contraindication for surgery [7]. The literature does report some cases treated successfully with thrombolytics [10] but most patients do need further treatment with anticoagulation [3] and there is a theoretical risk of lysed clots lodging in to pulmonary arteries and resulting in Pulmonary Embolism.

## 4. Conclusions

- Limited use of CVC for a short period, if CVC is to be used for a longer time, keeps high index of suspicion for CRAT.
- Early recognition and treatment prior to incidence of any complication related to CRAT can significantly reduce mortality.
- Recognize that CVC malfunction may be suggestive of CRAT.
- Early and proper patient education is the key in the management of CRAT.
- Close cardiorespiratory monitoring is needed while treating CRAT.
- RCTs needed to better delineate pathophysiologic as well as treatment aspects of this fatal complication and to determine outcome variables.
